# Quality of Life Differences for Primary Immunodeficiency Patients on Home SCIG versus IVIG

**DOI:** 10.1007/s10875-019-00705-5

**Published:** 2019-11-01

**Authors:** Christine Anterasian, Richard Duong, Peg Gruenemeier, Carol Ernst, Jessica Kitsen, Bob Geng

**Affiliations:** 1 Division of Allergy/Immunology, University of California, San Diego, San Diego, CA 92123 USA; 2 Diplomat Pharmacy, Flint, MI 48507 USA

**Keywords:** Primary immunodeficiency, antibody deficiency, quality of life, health-related quality of life, subcutaneous immunoglobulin, intravenous immunoglobulin, SCIG, IVIG

## Abstract

**Background:**

Patients with primary immunodeficiency disease (PIDD) and antibody deficiency require lifelong immunoglobulin replacement therapy. While both subcutaneous immunoglobulin (SCIG) and intravenous immunoglobulin (IVIG) replacement therapy are effective in preventing infection, patients with PIDD still experience worse health-related quality of life (hrQOL) outcomes.

**Objective:**

Assess differences in hrQOL for PIDD patients receiving home SCIG versus IVIG.

**Methods:**

SF-36 surveys were administered by a specialty pharmacy to 630 PIDD patients receiving home SCIG and IVIG at baseline and then every 3 months between 2014 and 2016. Results were analyzed using two-sample *t* tests and linear mixed effects model. Analysis was repeated for different age categories and trended over time.

**Results:**

Patients receiving SCIG reported statistically significant higher energy fatigue scores (+ 9 points, *p* < 0.001) but lower perceived role limitations due to physical health scores (− 14 points, *p* < 0.001). These differences were only observed in patients > 36 years of age. There were no differences in the composite SF-36 score for patients receiving SCIG versus IVIG (+ 1, *p* = 0.66). Immunoglobulin-naïve patients all improved their hrQOL, but a larger improvement was seen in those initiating SCIG versus IVIG.

**Conclusion:**

Patients with PIDD on home IVIG versus SCIG have similar composite hrQOL scores as measured by the SF-36. In the adult population, initiating immunoglobulin replacement with SCIG may result in more hrQOL improvement compared with IVIG, although personal preferences should also be considered.

**Clinical Implications:**

Patients with PIDD on home IVIG versus SCIG have similar composite health-related quality of life scores as measured by the SF-36.

**Capsule Summary:**

Patients with primary immune-deficiency on home IVIG versus SCIG have similar composite health-related quality of life scores as measured by the SF-36. Personal preferences are important in deciding whether to treat with IVIG or SCIG.

**Electronic supplementary material:**

The online version of this article (10.1007/s10875-019-00705-5) contains supplementary material, which is available to authorized users.

## Background

Primary immunodeficiency disease (PIDD) is a group of over 300 diseases characterized by defects within the immune system. Immunoglobulin replacement is a mainstay of therapy for the majority of patients with antibody deficiencies. Immunoglobulin replacement is administered by the intravenous route (IVIG) or subcutaneous route (SCIG) to return immunoglobulin levels to physiologic levels [1–3].

IVIG is administered approximately once per month by a licensed healthcare professional either at an infusion center or through home healthcare with an infusion nurse. SCIG is a newer treatment that has been shown to be safe [4, 5] and efficacious [6, 7] with a favorable safety profile [8–11] compared with IVIG. SCIG is able to achieve similar average Ig levels but with less variation between treatments [5, 12]. SCIG can also be self-administered, thus eliminating the need for monthly home health or infusion center visits [4, 13, 14] and allowing patients more flexibility over their schedules.

Many studies have looked at the clinical benefit of these two treatments, and while both are effective in preventing infection [6], studies suggest that these patients still have lower health-related quality of life with regards to both mental and physical functioning [15, 16]. Some studies demonstrate improvement in hrQOL for PIDD patients after switching from IVIG to SCIG [7, 9, 17, 18]. However, these studies are often small and do not address trends in hrQOL among different age groups and over time and often do not control for location of therapy administration.

The SF-36 survey is a frequently used tool to measure patient hrQOL across 8 different domains, including physical functioning, bodily pain, role limitations due to physical health problems, role limitations due to personal or emotional problems, emotional well-being, social functioning, energy/fatigue, and general health perceptions. This study analyzes prospectively collected SF-36 surveys administered to patients with PIDD with the primary aim of assessing whether there are differences in hrQOL scores across the 8 different domains of the SF-36 for patients on SCIG vs IVIG. Secondary aims include assessing if patient age impacts the relationship between hrQOL and Ig formulation and if time impacts the relationship between hrQOL and Ig formulation across SF-36 domains. An exploratory tertiary aim involves assessing changes in SF-36 scores across the 8 domains in immunoglobulin-naïve patients before and after initiating treatment with IVIG versus SCIG.

## Methods

**Fig. 1:**
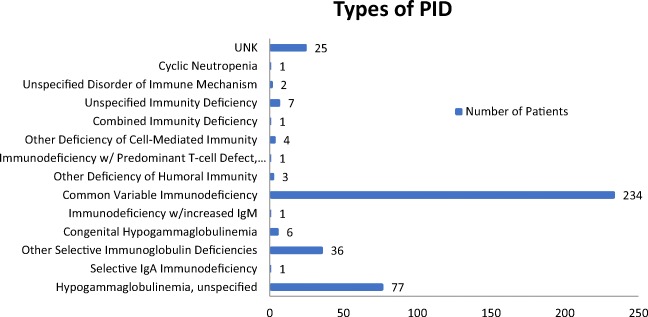
Types of PID of 399 of the patients available through the ICD 9/10 codes

**Fig. 2:**
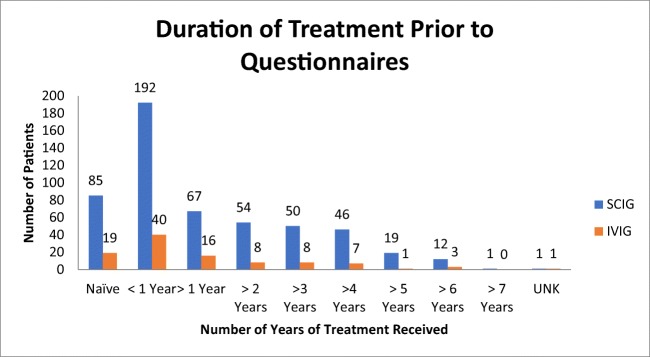
Duration of SCIG or IVIG treatment prior to the observational period

**Fig. 3:**
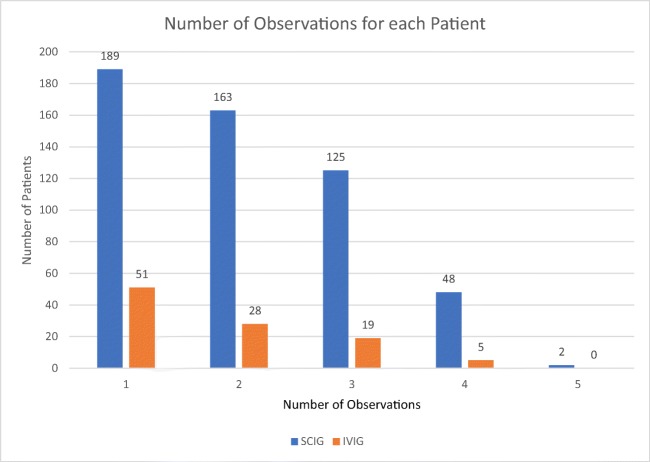
Number of observations captured for each patient within each treatment group

This study was designed as a retrospective data analysis. As part of standard care, SF-36 surveys were administered by a specialty pharmacy via telephone to patients with predominately antibody deficiency (Fig. 1) receiving either home IVIG or SCIG from 2014 to 2016. Each patient had SF-36 results collected every 3 months as available. Most patients were continuing treatment; however, 104 patients had a baseline questionnaire prior to starting treatment. Of the patients continuing SCIG treatment, 192 had started treatment within the year of the first questionnaire, 249 patients had been receiving treatment 1–7 years prior to collection, and 1 was unknown. For the patients continuing IVIG treatment, 40 had started within the year of the first questionnaire, 43 had been receiving treatment 1–6 years prior to collection, and 1 was unknown (Fig. 2). At the time of data collection, the questionnaire was collected as a part of the pharmacy’s routine clinical care, and therefore, the questionnaires were aware of the route of therapy the respondents where on. It was only after the data had been collected that the data was deidentified and shared with the PI of this study for statistical analysis. From the records covering this time period, none of the patients had switched therapies, and the amount of questionnaires completed by each subject ranged from 1 to 5 (Fig. 3).

### Statistics

We used a two-sample *t* test to compare scores averaged within subject between the IVIG and SCIG groups, and to compare age distributions with Fisher’s exact test. We also used a linear mixed effects model to leverage information from repeated measures within each subject, accounting for within subject correlation with a random intercept and including fixed effects for SCIG vs IVIG group. This analysis is repeated within each age category, and a linear mixed model accounting for repeated measures within subject and with group and age category interactions is built to test whether a group has heterogeneous effects across ages. Overall significance of this interaction was determined by a conditional *F* test. *t* tests were used to compare average score before and after initiation with immunoglobulin replacement product.

## Results

**Fig. 4:**
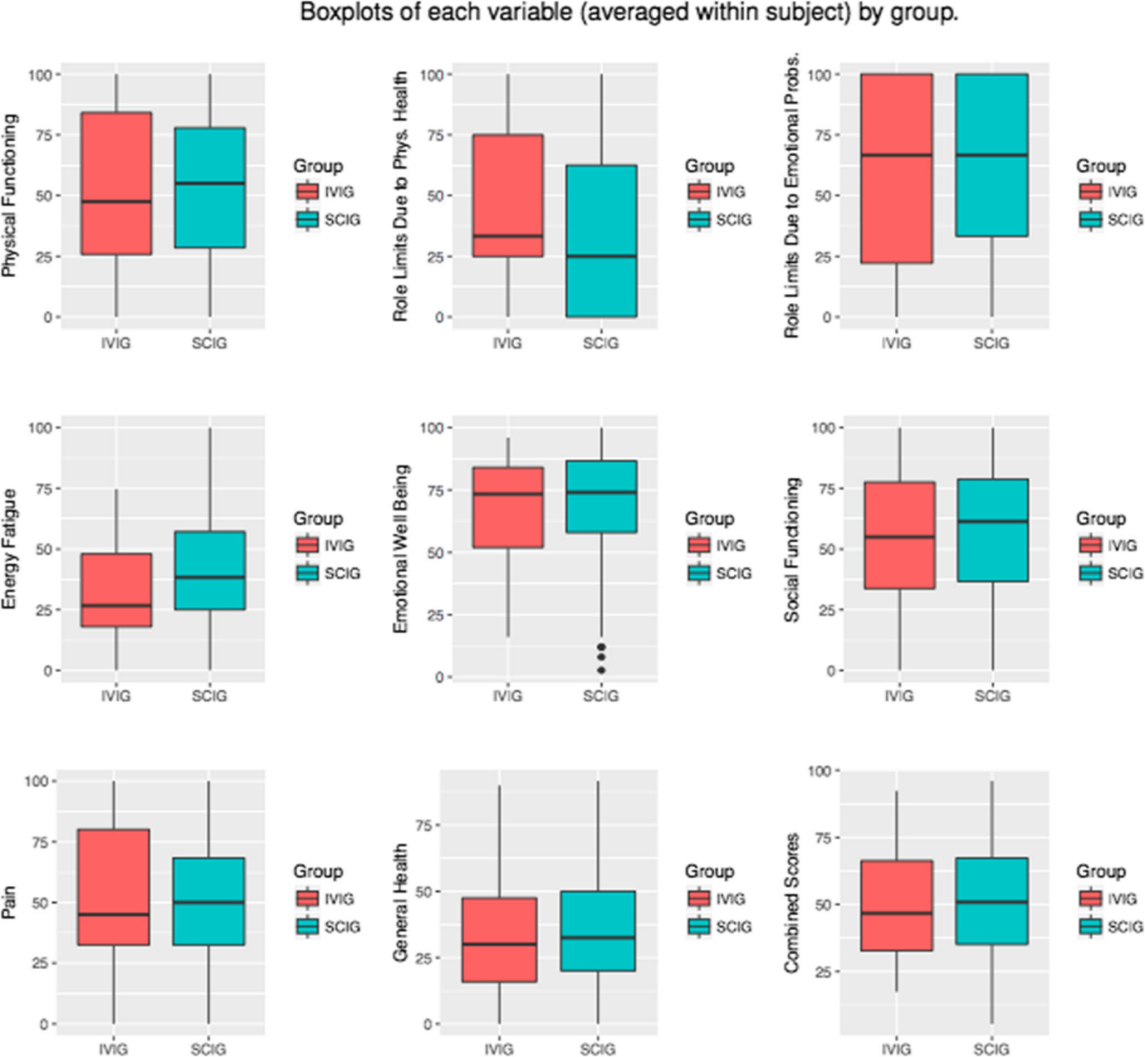
Boxplots of each outcome by IVIG versus SCIG

SF-36 results from 630 patients with PID were analyzed. To investigate differences in quality of life scores for patients on home SCIG versus IVIG across the 8 domains of the SF-36 and using a composite score, we first used a two-sample *t* test to compare scores averaged within subject. We found that patients on SCIG reported statistically significant higher scores in energy fatigue (+ 9 points, *p* < 0.001) and lower scores in perceived role limitations due to physical health (− 14 points, *p* < 0.001) (Table 1). There were no statistically significant differences in the 6 other domains or in the combined SF-36 score. We then used a linear mixed effects regression model to leverage information from multiple SF-36 results within each subject including only a fixed effect for whether the subject was on SCIG vs IVIG and again found a statistically significant higher reporting of energy fatigue scores (+ 9 points, *p* < 0.001) and lower reporting of perceived role limitations due to physical health scores (− 14 points, *p* < 0.001) (Fig. 4).

**Table 1:** Mean (SD) of scores averaged within each subject. Significance is determined by a two-sample *t* test

	Group			
	IVIG (*n* = 103)	SCIG (*n* = 527)	Overall (*n* = 630)	*p* value
Age group				
35 or younger	15 (14.6%)	48 (9.1%)	63 (10.0%)	0.223
36–60	38 (36.9%)	196 (37.2%)	234 (37.1%)	
Over 60	50 (48.5%)	283 (53.7%)	333 (52.9%)	
Physical functioning	53.15 (31.52)	53.05 (28.74)	53.06 (29.19)	0.975
Role limits due to physical health	49.13 (29.98)	34.71 (36.51)	37.06 (35.90)	< 0.001*
Role limits due to emotional problems	56.39 (39.25)	63.97 (36.23)	62.73 (36.81)	0.072
Energy fatigue	32.03 (19.40)	41.01 (22.65)	39.54 (22.38)	< 0.001*
Emotional well-being	68.22 (19.94)	70.49 (19.90)	70.12 (19.91)	0.292
Social functioning	55.33 (27.31)	58.79 (26.25)	58.23 (26.44)	0.238
Pain	53.48 (27.82)	51.77 (25.40)	52.05 (25.80)	0.564
General health	33.55 (21.31)	35.57 (19.29)	35.24 (19.63)	0.373
Combined scores	50.16 (21.16)	51.17 (21.21)	51.00 (21.19)	0.659

The asterisks denote statistically significant comparisons

Our secondary aim was to assess if there was a significant relationship between age, home SCIG vs IVIG, and quality of life as measured by the SF-36. Using the Fisher exact test, we found that there was no relationship between a patient’s age category (≤ 35 years old, 36–60 years old, > 60 years old) and the likelihood they were taking SCIG vs IVIG (*p* = 0.223) (Table 1). Using a subgroup analysis by patient age (Table 2), we found that patients ≤ 35 years old had no statistically significant differences in SF-36 hrQOL scores using both a *t* test of mean scores averaged within subject and a linear mixed regression model with only a SCIG versus IVIG fixed effect. When the same analysis was repeated for patients 36–60 years of age, patients on SCIG reported statistically significant higher energy fatigue scores (*t* test + 9, *p* = 0.011; regression model + 8, *p* = 0.032) and lower scores in both perceived role limitations due to physical health (*t* test − 20.6, *p* < 0.001; model − 20.5, *p* = 0.002) and physical functioning (*t* test − 11, *p* = 0.036; model − 11, *p* < 0.001). Patients > 60 years of age and on SCIG likewise reported higher scores for energy fatigue (*t* test + 9.6, *p* = 0.002; model + 9.6, *p* < 0.001) and lower scores for perceived role limitations due to physical health (*t* test − 11, *p* = 0.012; model − 11.5, *p* < 0.001) but interestingly reported higher physical functioning scores in the regression model only (+ 9, *p* < 0.039). Results for all other domains and combined SF-36 scores were statistically insignificant.

**Table 2:** Mean (SD) of scores averaged within each subject with significance is determined by a two-sample *t* test and linear mixed model results using only SCIG vs IVIG group with a random intercept to account for within subject variability stratified by age category

	Age < 35					Age 36–60					Age > 60				
	Avg (SD) IVIG score *n* = 15	Avg (SD) SCIG score *n* = 48	*t* test *p* value	SCIG model estimate	Model *p* value	Avg (SD) IVIG score *n* = 38	Avg (SD) SCIG score *n* = 196	*p* value	SCIG model estimate	*p* value	Avg (SD) IVIG score *n* = 50	Avg (SD) SCIG score *n* = 283	*t* test *p* value	SCIG model estimate	Model *p* value
Physical functioning	65 (32)	72 (30)	0.471	7.1	0.429	64 (29)	52 (28.5)	0.036*	− 11.2	0.028*	41 (29)	50 (28)	0.054	8.9	0.039
Role limits due to physical health	52 (36)	47 (43)	0.648	− 2.1	0.862	52 (31)	32 (37)	< 0.001*	− 20.5	0.002*	35 (34)	35 (34)	0.012*	− 11.5	0.025*
Role limits due to emotional problems	56 (43)	62 (41)	0.68	8	0.496	54 (40)	60 (37)	0.447	5.1	0.448	67 (34)	67 (34)	0.109	8.4	0.116
Energy fatigue	38 (22)	46 (48)	0.252	8.9	0.265	27 (19)	36 (22)	0.011*	8.2	0.032*	44 (22)	44 (22)	0.002*	9.5	0.004*
Emotional well-being	66 (23)	70 (21)	0.602	4.1	0.519	66 (22)	65 (21)	0.862	− 0.4	0.92	75 (18)	75 (18)	0.172	3.6	0.188
Social functioning	52 (29)	63 (28)	0.181	12.3	0.15	56 (28)	54 (28)	0.581	− 2.5	0.616	62 (24)	62 (24)	0.151	5.4	0.14
Pain	63 (27)	62 (28)	0.92	− 0.3	0.966	53 (27)	49 (26)	0.374	− 4.3	0.365	52 (24)	52 (24)	0.797	1.3	0.74
General health	33 (19)	37 (23)	0.49	4.6	0.494	34 (22)	31 (18)	0.326	− 4.1	0.234	39 (19)	39 (19)	0.098	5.8	0.051
Combined score	53 (23)	57 (25)	0.548	4.9	0.514	51 (22)	47 (22)	0.35	− 3.7	0.35	53 (19)	53 (19)	0.199	4.1	0.178

The asterisks denote statistically significant comparisons

**Fig. 5:**
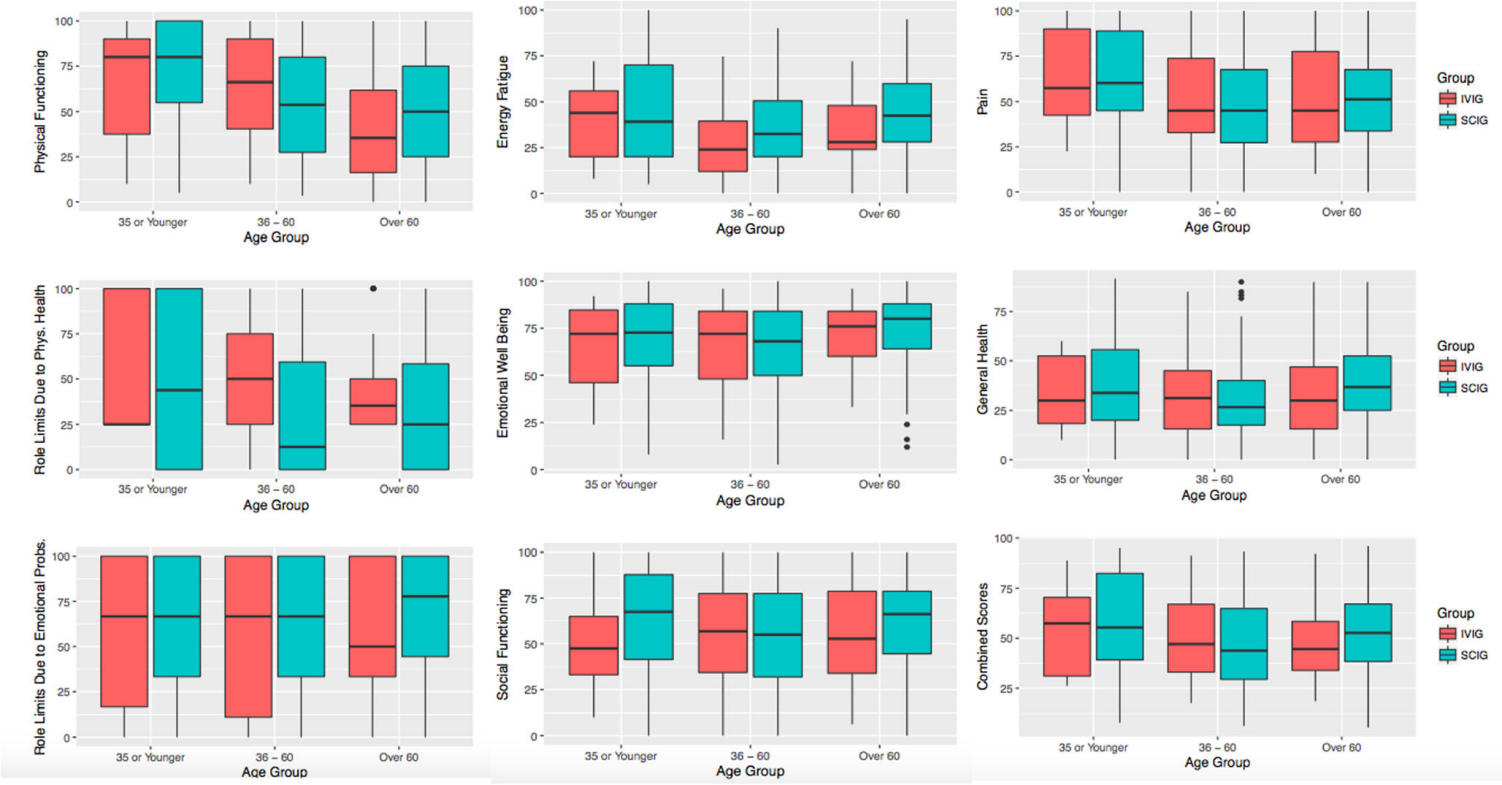
Boxplots of each outcome by IVIG/SCIG and age group

To investigate whether being on SCIG versus IVIG has a heterogeneous effect across ages, we built a model to allow for interactions between these variables. We found a significant interaction between age groups and SCIG versus IVIG in physical functioning, indicating that the large negative effect SCIG had in the 36–59-year-old age group was significantly different from the effects seen in our other age groups (*p* = 0.008) (Fig. 5). There were no significant interactions in other domains or with a combined SF-36 score.

Accounting for differences in SF-36 scores across time in the models (with or without allowing for interactions between time and SCIG versus IVIG group) did not reveal any statistically significant interactions except for physical functioning where being on IVIG resulted in a slight upward trend of SF-36 scores over time which was not seen in the SCIG group (*p* = 0.045).

An exploratory tertiary aim of this study involved analyzing differences in hrQOL as measured by SF-36 for patients before and after starting on home immunoglobulin replacement. A total of 104 subjects had baseline SF-36 scores obtained prior to starting immunoglobulin replacement, 11 of whom were started on IVIG and 93 of whom were started on SCIG. Patients who were started on IVIG showed statistically significant improvement in 5 of 8 SF-36 domains (physical functioning, energy, emotional well-being, social functioning, and general health), whereas patients started on SCIG showed statistically significant improvement in all 8 domains (Table 3). We were unable to compare differences in the magnitude of improvement between the IVIG and SCIG groups due to the small sample size of the IVIG group.

**Table 3:** Mean scores before and after initiation of immunoglobulin replacement produce with significance determined by two-sample *t* test

	IVIG (11 patients)				SCIG (93 patients)			
SF-36 domain	Baseline	Assessment	Difference	*p* value	Baseline	Assessment	Difference	*p* value
Physical functioning	46.4	59.6	13.2	0.002	56.2	63.1	6.9	0.001
Role limitations due to physical function	25	40.9	15.9	0.111	30.6	45.4	14.8	0.002
Role limits due to emotional	42.4	45.5	3.0	0.884	60.9	74.3	13.3	0.007
Energy and fatigue	30	57.3	27.3	0.001	38.2	48.7	10.4	< 0.001
Emotional well-being	59.3	76.7	17.5	0.001	71.1	75.7	4.7	0.021
Social functioning	39.8	62.1	22.3	0.005	54.5	66.2	11.7	< 0.001
Pain	60.5	70.1	10.5	0.218	54.1	60	5.8	0.030
General health	24.1	48.6	24.5	0.001	34.6	42.3	7.7	< 0.001

## Discussion

The current study reflects self-reported hrQOL scores for patients receiving home IVIG versus SCIG across the 8 different domains of the SF-36 survey. The data includes a total of 103 IVIG and 527 SCIG patients. The imbalance in subject population reflects the actual census of the patients being treated by the specialty pharmacy at the time that the data was analyzed. This imbalance did not influence the statistical validity of the overall results as determined by our statistician.

We show that patients with primary immunodeficiency receiving SCIG report higher hrQOL scores related to energy/fatigue but lower hrQOL scores related to perceived role limitations due to physical health compared with patients receiving IVIG. This results in no differences in the overall composite SF-36 score.

Most studies assessing hrQOL in PIDD patients receiving SCIG versus IVIG using the SF-36 show higher reported scores in patient receiving SCIG, although which domains are statistically significant varies. Similar to our findings, other studies [19–22] have demonstrated higher energy/fatigue in patients receiving SCIG. This makes physiologic sense as patients on SCIG have less “wear-off” effect due to better steady-state Ig levels [4] and less systemic side effects associated with the IVIG peak, including hemolytic anemia.

However, other studies [20, 21] demonstrated higher scores for role limitations due to physical health for patients on SCIG whereas our study reflects decreased hrQOL in this domain in patients > 35 years of age. At times, other studies have shown higher reported scores in general health [19–21], mental health [19, 22], and social functioning [22, 23] scores in patients receiving SCIG, whereas our study shows no significant difference in these domains.

One factor that may account for these discrepancies is that all patients in this cohort were receiving their IVIG or SCIG in the home setting, and thus, the results of our study are not biased by differences in SF-36 domains susceptible to changes based on location of administration [9, 22]. It is also likely differences in study design account for some of these discrepancies. Most studies in PIDD patients are structured so that hrQOL outcomes are measured before and after switching immunoglobulin replacement product (commonly IVIG>SCIG). These studies likely overestimate SCIG hrQOL improvement as the patients who choose to make this switch are already unsatisfied with their previous method of immunoglobulin replacement. Our study was observational and is not subject to this same bias. However, our study does not control for differences in baseline functional status between the patients on SCIG versus IVIG and thus the differences we captured in hrQOL may not all be related to method of immunoglobulin replacement.

In a similarly designed survey-based hrQOL study by Espanol [16] with an international cohort of 300 PIDD patients, those on SCIG had higher baseline hrQOL scores compared with those on IVIG as measured by the SF-12 and EuroQOL-5 Dimensions. In our study, while patients on SCIG had higher reported scores in energy/fatigue compared with those on IVIG, this was counterbalanced by lower reported scores in perceived role limitations due to physical functioning resulting in no net difference in hrQOL. It is possible that this discrepancy is due to study population and cultural differences as that cohort of patients was primarily European (only 31 Canadian, no American). Together, these results suggest that the decision whether to treat with SCIG versus IVIG should be individualized to account for patient preferences [24].

Patient education is a critical piece in improving hrQOL. Patients need to have realistic expectations for the benefits and challenges of each method so they can determine which approach is correct for them since there are tradeoffs to be made as reflected in the results of this study. Most patients prefer SCIG as it is home-based [14, 21], more convenient, allows them greater independence, and is thought to be better tolerated with fewer systemic reactions [25]. Due to flexibility in dosing frequency and scheduling [22, 26], patients can also achieve better steady-state immunoglobulin concentrations [4] resulting in fewer hospital days [25] and fewer missed days of work [27]. Lastly, self-infusion at home also decreases utilization of the healthcare system and, most importantly, empowers the patients and promotes their responsibility [9, 21]. However, some patients prefer IVIG and identify perceived inconvenience, concerns about adverse reactions at home, and apprehension over frequent needle sticks as their concerns [19, 28].

Espanol et al. [16] demonstrated that 76% of PIDD patients were satisfied with their current treatment in terms of SCIG versus IVIG, yet this population still experiences impaired hrQOL related to both mental and physical functioning [15]. Patients have identified immunoglobulin self-administration, home administration, shorter duration of administration, fewer needle sticks, and once per month administration as features that improve their hrQOL [16, 29].

Regardless of method of administration, the initiation of immunoglobulin replacement therapy prolongs survival, reduces morbidity, and results in an improvement in hrQOL. In our study immunoglobulin-naïve patients who initiated SCIG replacement therapy demonstrated statistically significant improvement in more domains of the SF-36 than the IVIG group. This is consistent with the majority of the literature [16, 22, 30] including a metaanalysis [25] of 47 different studies involving 1484 patient cases which suggests that most patients experience higher hrQOL outcomes when receiving SCIG compared with IVIG.

In determining if our immunoglobulin-naïve patients experienced a clinically/socially meaningful difference aside from statistics, we were able to compare their difference values with the minimally important difference (MID) values as referenced by Routes et al. [31] We found that SCIG patients met the MID in every domain except the emotional well-being and IVIG met every domain except the role limitations due to emotional problems. The clinically important difference for each group was determined as 4.3 for physical functioning, 4.0 for role limitations due to physical health, 5.5 for bodily pain, 7.0 for general health, 6.7 for energy/fatigue, 6.2 for social functioning, 4.6 for role limitations due to emotional well-being, and 6.7 for mental health [31].

This study was the first to assess if there were differences in hrQOL scores for PIDD patients receiving SCIG versus IVIG according to age. Using composite SF-36 scores, there were no overall differences in hrQOL according to age. However, patients > 36 and on SCIG reported statistically significant higher energy fatigue scores and lower perceived role limitations due to physical health scores. Patients over 60 on SCIG actually reported higher scores in their physical functioning when on SCIG versus IVIG despite perceiving increased role limitations due to physical health. This suggests that despite perceiving lower health, they actually have an increased ability to accomplish their activities of daily living when on SCIG versus IVIG.

Similarly, this study was the first to assess if there were differences in hrQOL scores over time for PIDD patients receiving SCIG versus IVIG. We did not observe any significant trends over time in hrQOL scores for patients receiving SCIG versus IVIG. Although patients receiving IVIG reported statistically significant (*p* = 0.045) higher scores in physical functioning scores over time that was not seen in SCIG patients, it is unlikely this is of true clinical significance. In a 6-year longitudinal study [32], hrQOL declined over time in patients with common-variable immune-deficiency. Although we did not see a similar trend, this is likely because of the significantly shorter follow-up interval.

It is important to note that patient-perceived QOL is distinct from treatment satisfaction, which was not measured in the analyses in this study. It would be helpful in future studies to examine the correlation between perceived QOL and treatment satisfaction scores. The goal of this study is descriptive and not necessarily prescriptive on the determination of choice of therapy route. The results highlight differences in perceived QOLs but not necessarily preference of one therapy over another from the patient’s perspective. In order to better assess that, it would be important to directly measure satisfaction scores for both the treatment modality as well as overall perception of their state of health.

There are many limitations for this study. As previously mentioned, this is an observational study and thus does not captures differences in hrQOL for individual patients when switching between SCIG and IVIG and may be biased by baseline differences in each population of patients. Furthermore, all patients received their immunoglobulin replacement product from only one specialty pharmacy and differences in administration logistics specific to that institution may have influenced hrQOL outcomes. Changes based on age may be confounded by unmeasured variables, such as infection rate, hospitalization, and comorbid diseases. Lastly, because the SF-36 was phone administered, there is the potential for recall bias, response bias, and question misunderstanding.

## Electronic supplementary material

ESM 1 (PDF 1834 kb)

## Abbreviations

hrQOL: Health-related quality of life
IVIG: Intravenous immunoglobulin
SCIG: Subcutaneous immunoglobulin
PIDD: Primary immunodeficiency disease
