# Supplementary material for: Quality of Life Differences for Primary Immunodeficiency Patients on Home SCIG versus IVIG
Source: J Clin Immunol. 2019 Nov 1;39(8):814–22. doi: 10.1007/s10875-019-00705-5 (PMC6863943; doi:10.1007/s10875-019-00705-5)
Supplement: Supplementary file 1 — (PDF 1834 kb) [file 10875_2019_705_MOESM1_ESM.pdf]

RAND CORPORATION HEALTH CARE

36-Item Short Form Survey Instrument (SF-36)

RAND 36-Item Health Survey 1.0 Questionnaire Items

Choose one option for each questionnaire item.

1. In general, would you say your health is:

- 1 - Excellent
- 2 - Very good
- 3 - Good
- 4 - Fair
- 5 - Poor

---

2. **Compared to one year ago**, how would you rate your health in general **now**?

- 1 - Much better now than one year ago
- 2 - Somewhat better now than one year ago
- 3 - About the same
- 4 - Somewhat worse now than one year ago
- 5 - Much worse now than one year ago

The following items are about activities you might do during a typical day. Does **your health now limit you** in these activities? If so, how much?

	Yes, limited a lot	Yes, limited a little	No, not limited at all
3. **Vigorous activities**, such as running, lifting heavy objects, participating in strenuous sports	1	2	3
4. **Moderate activities**, such as moving a table, pushing a vacuum cleaner, bowling, or playing golf	1	2	3
5. Lifting or carrying groceries	1	2	3
6. Climbing **several** flights of stairs	1	2	3
7. Climbing **one** flight of stairs	1	2	3
8. Bending, kneeling, or stooping	1	2	3
9. Walking **more than a mile**	1	2	3
10. Walking **several blocks**	1	2	3
11. Walking **one block**	1	2	3
12. Bathing or dressing yourself	1	2	3

During the **past 4 weeks**, have you had any of the following problems with your work or other regular daily activities **as a result of your physical health**?

	Yes	No
13. Cut down the **amount of time** you spent on work or other activities	1	2
14. **Accomplished less** than you would like	1	2
15. Were limited in the **kind** of work or other activities	1	2
16. Had **difficulty** performing the work or other activities (for example, it took extra effort)	1	2

During the **past 4 weeks**, have you had any of the following problems with your work or other regular daily activities **as a result of any emotional problems** (such as feeling depressed or anxious)?

	Yes	No
17. Cut down the **amount of time** you spent on work or other activities	1	2
18. **Accomplished less** than you would like	1	2
19. Didn't do work or other activities as **carefully** as usual	1	2

20. During the **past 4 weeks**, to what extent has your physical health or emotional problems interfered with your normal social activities with family, friends, neighbors, or groups?

- 1 - Not at all
- 2 - Slightly
- 3 - Moderately
- 4 - Quite a bit
- 5 - Extremely

21. How much **bodily** pain have you had during the **past 4 weeks**?

- 1 - None
- 2 - Very mild
- 3 - Mild
- 4 - Moderate
- 5 - Severe
- 6 - Very severe

22. During the **past 4 weeks**, how much did **pain** interfere with your normal work (including both work outside the home and housework)?

- 1 - Not at all
- 2 - A little bit
- 3 - Moderately
- 4 - Quite a bit
- 5 - Extremely

These questions are about how you feel and how things have been with you **during the past 4 weeks**. For each question, please give the one answer that comes closest to the way you have been feeling.

How much of the time during the **past 4 weeks**...

	All of the time	Most of the time	A good bit of the time	Some of the time	A little of the time	None of the time
23. Did you feel full of pep?	1	2	3	4	5	6
24. Have you been a very nervous person?	1	2	3	4	5	6
25. Have you felt so down in the dumps that nothing could cheer you up?	1	2	3	4	5	6
26. Have you felt calm and peaceful?	1	2	3	4	5	6
27. Did you have a lot of energy?	1	2	3	4	5	6
28. Have you felt downhearted and blue?	1	2	3	4	5	6
29. Did you feel worn out?	1	2	3	4	5	6
30. Have you been a happy person?	1	2	3	4	5	6
31. Did you feel tired?	1	2	3	4	5	6

32. During the **past 4 weeks**, how much of the time has **your physical health or emotional problems** interfered with your social activities (like visiting with friends, relatives, etc.)?

- 1 - All of the time
- 2 - Most of the time
- 3 - Some of the time
- 4 - A little of the time
- 5 - None of the time

How TRUE or FALSE is **each** of the following statements for you.

	Definitely true	Mostly true	Don't know	Mostly false	Definitely false
33. I seem to get sick a little easier than other people	1	2	3	4	5
34. I am as healthy as anybody I know	1	2	3	4	5
35. I expect my health to get worse	1	2	3	4	5
36. My health is excellent	1	2	3	4	5

ABOUT

The RAND Corporation is a research organization that develops solutions to public policy challenges to help make communities throughout the world safer and more secure, healthier and more prosperous. RAND is nonprofit, nonpartisan, and committed to the public interest.

RAND® is a registered trademark. Copyright © 1994-2019 RAND Corporation.

HEALTH CARE

36-Item Short Form Survey (SF-36) Scoring Instructions

Introduction

The RAND 36-Item Health Survey (Version 1.0) taps eight health concepts: physical functioning, bodily pain, role limitations due to physical health problems, role limitations due to personal or emotional problems, emotional well-being, social functioning, energy/fatigue, and general health perceptions. It also includes a single item that provides an indication of perceived change in health. These 36 items, presented here, are identical to the MOS SF-36 described in Ware and Sherbourne (1992). They were adapted from longer instruments completed by patients participating in the Medical Outcomes Study (MOS), an observational study of variations in physician practice styles and patient outcomes in different systems of health care delivery (Hays & Shapiro, 1992; Stewart, Sherbourne, Hays, et al., 1992).

Scoring Rules for the RAND 36-Item Health Survey (Version 1.0)

We recommend that responses be scored as described below. A somewhat different scoring procedure for the MOS SF-36 has been distributed by the International Resource Center for Health Care Assessment (located in Boston, MA). Because the scoring method described here (a simpler and more straightforward procedure) differs from that of the MOS SF-36, persons using this scoring method should refer to the instrument as RAND 36-Item Health Survey 1.0.

Scoring the RAND 36-Item Health Survey is a two-step process. First, precoded numeric values are recoded per the scoring key given in Table 1. Note that all items are scored so that a high score defines a more favorable health state. In addition, each item is scored on a 0 to 100 range so that the lowest and highest possible scores are 0 and 100, respectively. Scores represent the percentage of total possible score achieved. In step 2, items in the same scale are averaged together to create the 8 scale scores. Table 2 lists the items averaged together to create each scale. Items that are left blank (missing data) are not taken into account when calculating the scale scores. Hence, scale scores represent the average for all items in the scale that the respondent answered.

Example: Items 20 and 32 are used to score the measure of social functioning. Each of the two items has 5 response choices. However, a high score (response choice 5) on item 20 indicates the presence of limitations in social functioning, while a high score (response choice 5) on item 32 indicates the absence of limitations in social functioning. To score both items in the same direction, Table 1 shows that responses 1 through 5 for item 20 should be recoded to values of 100, 75, 50, 25, and 0, respectively. Responses 1 through 5 for item 32 should be recoded to values of 0, 25, 50, 75, and 100, respectively. Table 2 shows that these two recoded items should be averaged together to form the social functioning scale. If the respondent is missing one of the two items, the person's score will be equal to that of the non-missing item.

Table 3 presents information on the reliability, central tendency, and variability of the scales scored using this method.

References

1. Ware, J.E., Jr., & Sherbourne, C.D. “The MOS 36-Item Short-Form Health Survey (SF-36): I. Conceptual Framework and Item Selection,”. *Medical Care*, 30:473-483, 1992.
2. Hays, R.D., & Shapiro, M.F. “An Overview of Generic Health-Related Quality of Life Measures for HIV Research,” *Quality of Life Research*. 1:91-97, 1992.
3. Steward, A.L., Sherbourne, C., Hayes, R.D., et al. “Summary and Discussion of MOS Measures,” in A.L. Stewart & J.E. Ware (eds.), *Measuring Functioning and Well-Being: The Medical Outcome Study Approach* (pp. 345-371). Durham, NC: Duke University Press, 1992.

Table 1

Step 1: Recoding Items

**Item numbers**	**Change original response category ***	**To recoded value of:**
1, 2, 20, 22, 34, 36	1 →	100
	2 →	75
	3 →	50
	4 →	25
	5 →	0
3, 4, 5, 6, 7, 8, 9, 10, 11, 12	1 →	0
	2 →	50
	3 →	100
13, 14, 15, 16, 17, 18, 19	1 →	0
	2 →	100
21, 23, 26, 27, 30	1 →	100
	2 →	80
	3 →	60
	4 →	40
	5 →	20
	6 →	0
24, 25, 28, 29, 31	1 →	0
	2 →	20
	3 →	40
	4 →	60
	5 →	80
	6 →	100
32, 33, 35	1 →	0
	2 →	25
	3 →	50
	4 →	75
	5 →	100

* Precoded response choices as printed in the questionnaire.

Table 2

Step 2: Averaging Items to Form Scales

Scale	Number of items	After recoding per Table 1, average the following items
Physical functioning	10	3 4 5 6 7 8 9 10 11 12
Role limitations due to physical health	4	13 14 15 16
Role limitations due to emotional problems	3	17 18 19
Energy/fatigue	4	23 27 29 31
Emotional well-being	5	24 25 26 28 30
Social functioning	2	20 32
Pain	2	21 22
General health	5	1 33 34 35 36

Table 3

Reliability, Central Tendency, and Variability of Scales in the Medical Outcomes Study

Scale	Items	Alpha	Mean	SD
Physical functioning	10	0.93	70.61	27.42
Role functioning/physical	4	0.84	52.97	40.78
Role functioning/emotional	3	0.83	65.78	40.71
Energy/fatigue	4	0.86	52.15	22.39
Emotional well-being	5	0.90	70.38	21.97
Social functioning	2	0.85	78.77	25.43
Pain	2	0.78	70.77	25.46
General health	5	0.78	56.99	21.11
Health change	1	—	59.14	23.12

Note: Data is from baseline of the Medical Outcomes Study (N=2471), except for "Health change," which was obtained one year later.

ABOUT

The RAND Corporation is a research organization that develops solutions to public policy challenges to help make communities throughout the world safer and more secure, healthier and more prosperous. RAND is nonprofit, nonpartisan, and committed to

the public interest.

RAND® is a registered trademark. Copyright © 1994-2019 RAND Corporation.
